# Supplementary figures and images for: Topography influences diurnal and seasonal microclimate fluctuations in hilly terrain environments of coastal California
Source: PLoS One. 2024 Mar 29;19(3):e0300378. doi: 10.1371/journal.pone.0300378 (PMC10980203; doi:10.1371/journal.pone.0300378)

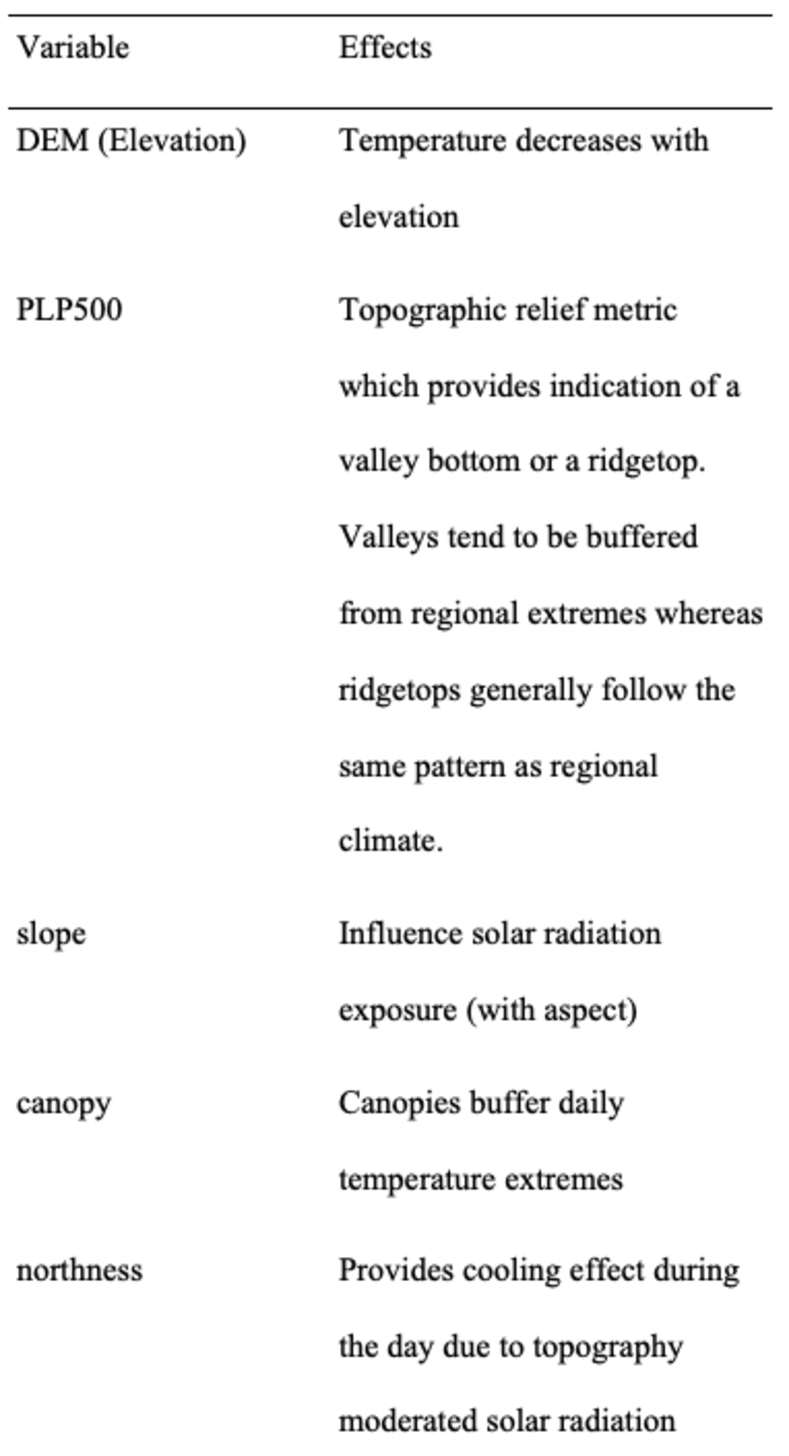

Supplement: S1 Appendix — Appendix A. Fig A.1. Fig A.1: Regression of climate variables against PC1 of physiographic space. Dominant variable explaining PC1 is DEM (elevation). Fig A.2. Fig A.2: Regression of climate variables against PC2 of physiographic space. Dominant variables explaining PC2 are canopy, PLP500 and northness. Fig A.3. Fig A.3: Custom radiation shield. Fig A.4. Fig A.4: Cold-air pooling evident on the northwest corner Pepperwood Preserve. (a) Mean minimum temperatures highlight inversion. (b) Season fluctuation (summer mean–winter mean) pronounced in the upper northwest corner of the Pepperwood Preserve, possibly part of larger cold-air pooling phenomena. Table. A.1. Table A.1 - RDA results showing climate space (only seasonal metrics) when constrained against physiographic space. Variables in bold are statistically significant (p < 0.05). Table. A.2. Table A.2 - Physiographic predictor variables and their effects. Appendix B. Fig B.1. Fig B.1: Mean temperature comparisons between HOBOs and an open site (Pepperwood Weather Station). Fig B.2. Fig B.2: Mean diurnal fluctuation comparisons between HOBOs and an open site (Pepperwood Weather Station) colored by elevation. Fig B.3. Fig B.3: Average annual temperature of each HOBO against the elevation (R2 = 0.23, p-value < 0.001). Fig B.4. Fig B.4: Pepperwood Preserve with 50 study sites numbered. (ZIP) [file pone.0300378.s001.zip › TableA2.tif]

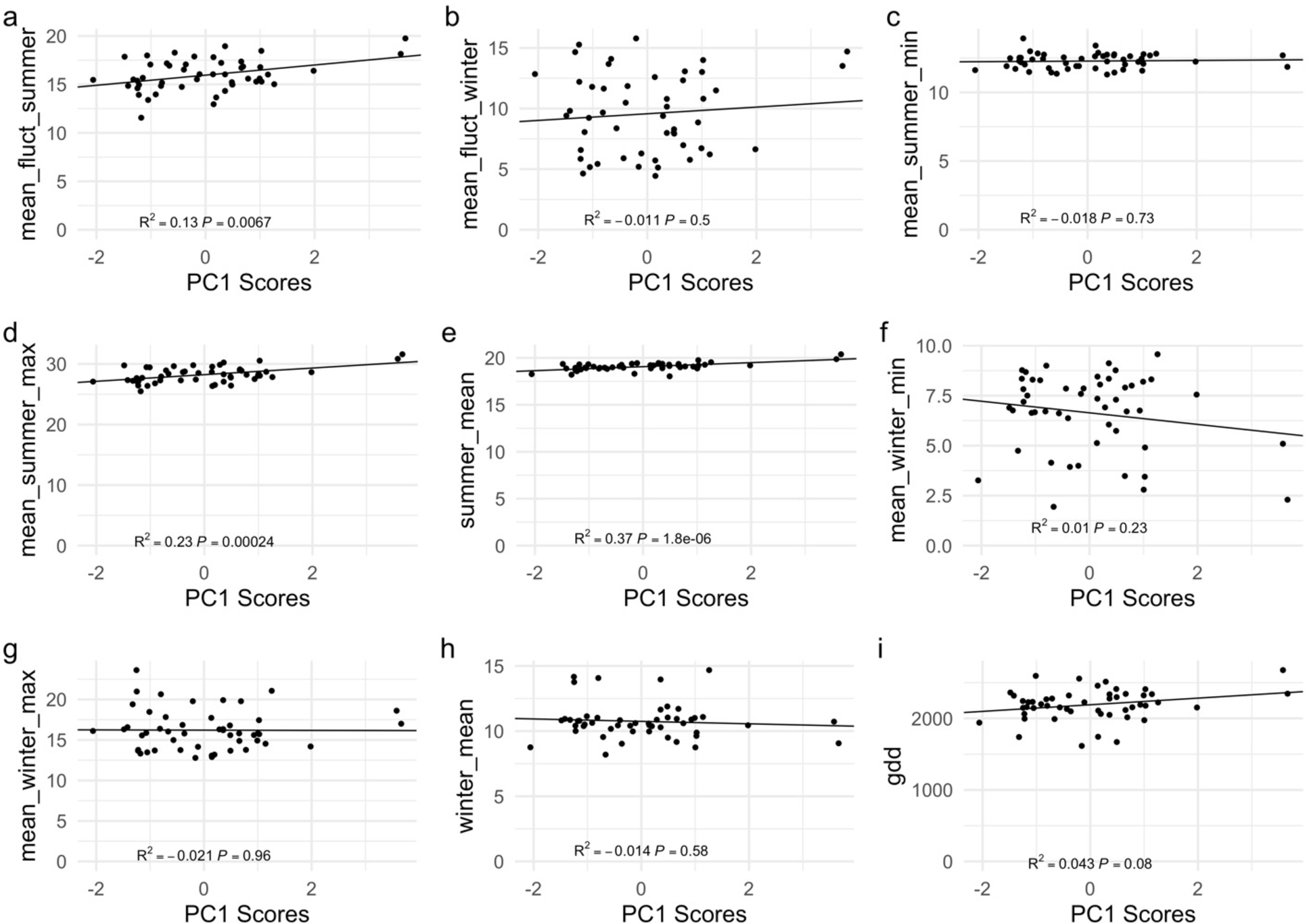

Supplement: S1 Appendix — Appendix A. Fig A.1. Fig A.1: Regression of climate variables against PC1 of physiographic space. Dominant variable explaining PC1 is DEM (elevation). Fig A.2. Fig A.2: Regression of climate variables against PC2 of physiographic space. Dominant variables explaining PC2 are canopy, PLP500 and northness. Fig A.3. Fig A.3: Custom radiation shield. Fig A.4. Fig A.4: Cold-air pooling evident on the northwest corner Pepperwood Preserve. (a) Mean minimum temperatures highlight inversion. (b) Season fluctuation (summer mean–winter mean) pronounced in the upper northwest corner of the Pepperwood Preserve, possibly part of larger cold-air pooling phenomena. Table. A.1. Table A.1 - RDA results showing climate space (only seasonal metrics) when constrained against physiographic space. Variables in bold are statistically significant (p < 0.05). Table. A.2. Table A.2 - Physiographic predictor variables and their effects. Appendix B. Fig B.1. Fig B.1: Mean temperature comparisons between HOBOs and an open site (Pepperwood Weather Station). Fig B.2. Fig B.2: Mean diurnal fluctuation comparisons between HOBOs and an open site (Pepperwood Weather Station) colored by elevation. Fig B.3. Fig B.3: Average annual temperature of each HOBO against the elevation (R2 = 0.23, p-value < 0.001). Fig B.4. Fig B.4: Pepperwood Preserve with 50 study sites numbered. (ZIP) [file pone.0300378.s001.zip › FigA1.tif]

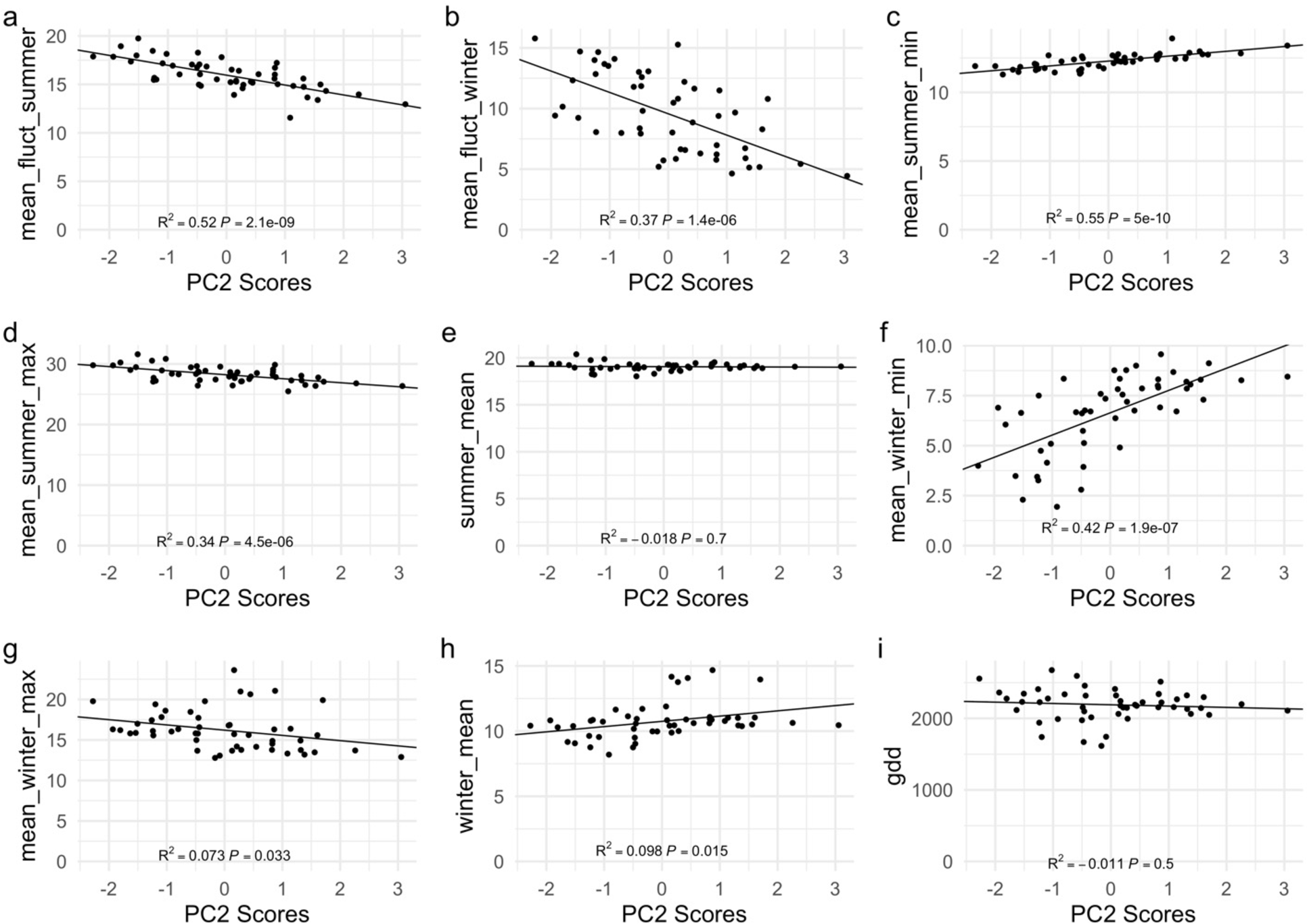

Supplement: S1 Appendix — Appendix A. Fig A.1. Fig A.1: Regression of climate variables against PC1 of physiographic space. Dominant variable explaining PC1 is DEM (elevation). Fig A.2. Fig A.2: Regression of climate variables against PC2 of physiographic space. Dominant variables explaining PC2 are canopy, PLP500 and northness. Fig A.3. Fig A.3: Custom radiation shield. Fig A.4. Fig A.4: Cold-air pooling evident on the northwest corner Pepperwood Preserve. (a) Mean minimum temperatures highlight inversion. (b) Season fluctuation (summer mean–winter mean) pronounced in the upper northwest corner of the Pepperwood Preserve, possibly part of larger cold-air pooling phenomena. Table. A.1. Table A.1 - RDA results showing climate space (only seasonal metrics) when constrained against physiographic space. Variables in bold are statistically significant (p < 0.05). Table. A.2. Table A.2 - Physiographic predictor variables and their effects. Appendix B. Fig B.1. Fig B.1: Mean temperature comparisons between HOBOs and an open site (Pepperwood Weather Station). Fig B.2. Fig B.2: Mean diurnal fluctuation comparisons between HOBOs and an open site (Pepperwood Weather Station) colored by elevation. Fig B.3. Fig B.3: Average annual temperature of each HOBO against the elevation (R2 = 0.23, p-value < 0.001). Fig B.4. Fig B.4: Pepperwood Preserve with 50 study sites numbered. (ZIP) [file pone.0300378.s001.zip › FigA2.tif]

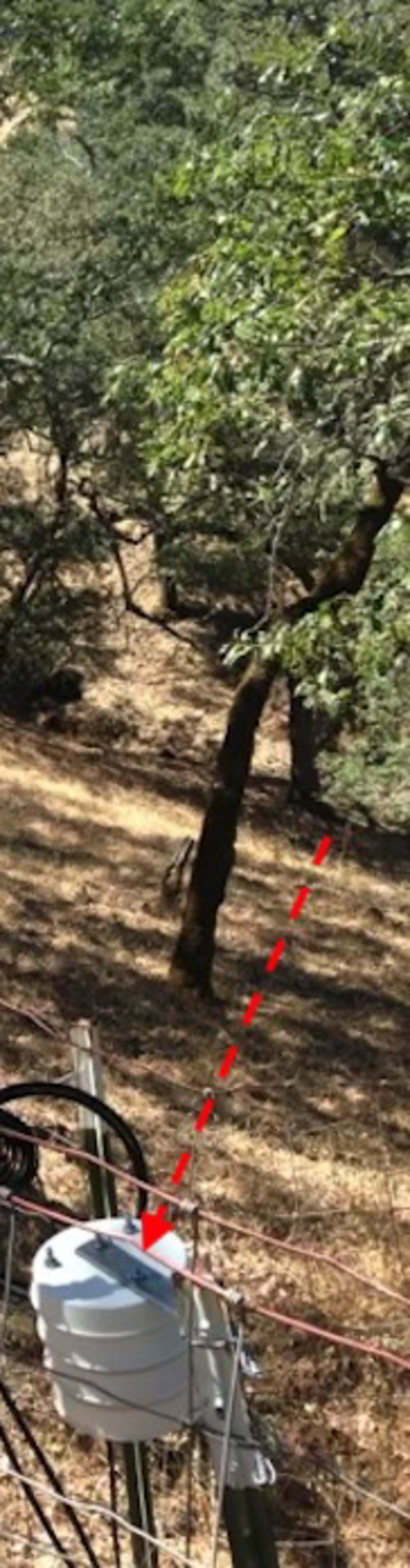

Supplement: S1 Appendix — Appendix A. Fig A.1. Fig A.1: Regression of climate variables against PC1 of physiographic space. Dominant variable explaining PC1 is DEM (elevation). Fig A.2. Fig A.2: Regression of climate variables against PC2 of physiographic space. Dominant variables explaining PC2 are canopy, PLP500 and northness. Fig A.3. Fig A.3: Custom radiation shield. Fig A.4. Fig A.4: Cold-air pooling evident on the northwest corner Pepperwood Preserve. (a) Mean minimum temperatures highlight inversion. (b) Season fluctuation (summer mean–winter mean) pronounced in the upper northwest corner of the Pepperwood Preserve, possibly part of larger cold-air pooling phenomena. Table. A.1. Table A.1 - RDA results showing climate space (only seasonal metrics) when constrained against physiographic space. Variables in bold are statistically significant (p < 0.05). Table. A.2. Table A.2 - Physiographic predictor variables and their effects. Appendix B. Fig B.1. Fig B.1: Mean temperature comparisons between HOBOs and an open site (Pepperwood Weather Station). Fig B.2. Fig B.2: Mean diurnal fluctuation comparisons between HOBOs and an open site (Pepperwood Weather Station) colored by elevation. Fig B.3. Fig B.3: Average annual temperature of each HOBO against the elevation (R2 = 0.23, p-value < 0.001). Fig B.4. Fig B.4: Pepperwood Preserve with 50 study sites numbered. (ZIP) [file pone.0300378.s001.zip › FigA3.tif]

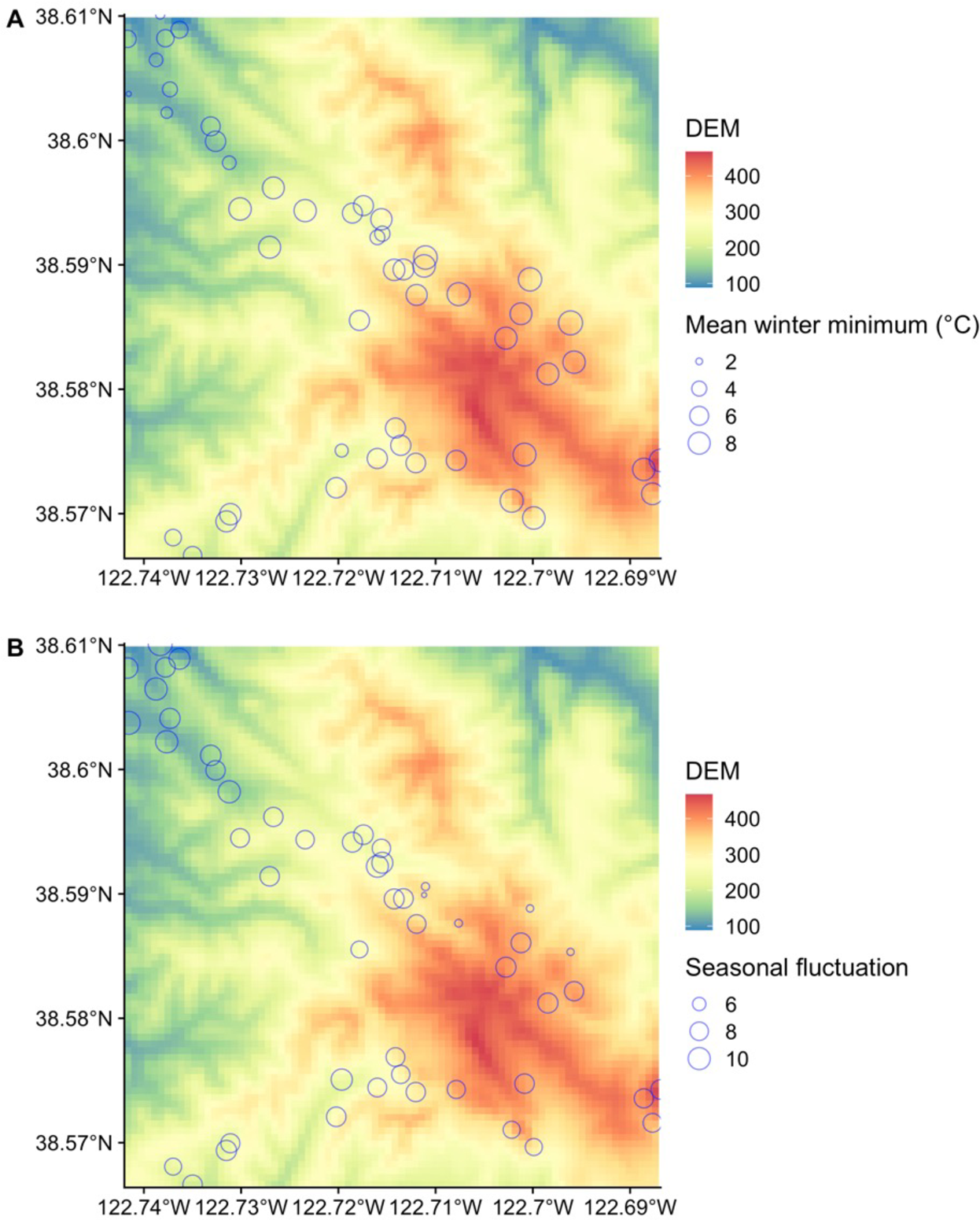

Supplement: S1 Appendix — Appendix A. Fig A.1. Fig A.1: Regression of climate variables against PC1 of physiographic space. Dominant variable explaining PC1 is DEM (elevation). Fig A.2. Fig A.2: Regression of climate variables against PC2 of physiographic space. Dominant variables explaining PC2 are canopy, PLP500 and northness. Fig A.3. Fig A.3: Custom radiation shield. Fig A.4. Fig A.4: Cold-air pooling evident on the northwest corner Pepperwood Preserve. (a) Mean minimum temperatures highlight inversion. (b) Season fluctuation (summer mean–winter mean) pronounced in the upper northwest corner of the Pepperwood Preserve, possibly part of larger cold-air pooling phenomena. Table. A.1. Table A.1 - RDA results showing climate space (only seasonal metrics) when constrained against physiographic space. Variables in bold are statistically significant (p < 0.05). Table. A.2. Table A.2 - Physiographic predictor variables and their effects. Appendix B. Fig B.1. Fig B.1: Mean temperature comparisons between HOBOs and an open site (Pepperwood Weather Station). Fig B.2. Fig B.2: Mean diurnal fluctuation comparisons between HOBOs and an open site (Pepperwood Weather Station) colored by elevation. Fig B.3. Fig B.3: Average annual temperature of each HOBO against the elevation (R2 = 0.23, p-value < 0.001). Fig B.4. Fig B.4: Pepperwood Preserve with 50 study sites numbered. (ZIP) [file pone.0300378.s001.zip › FigA4.tif]

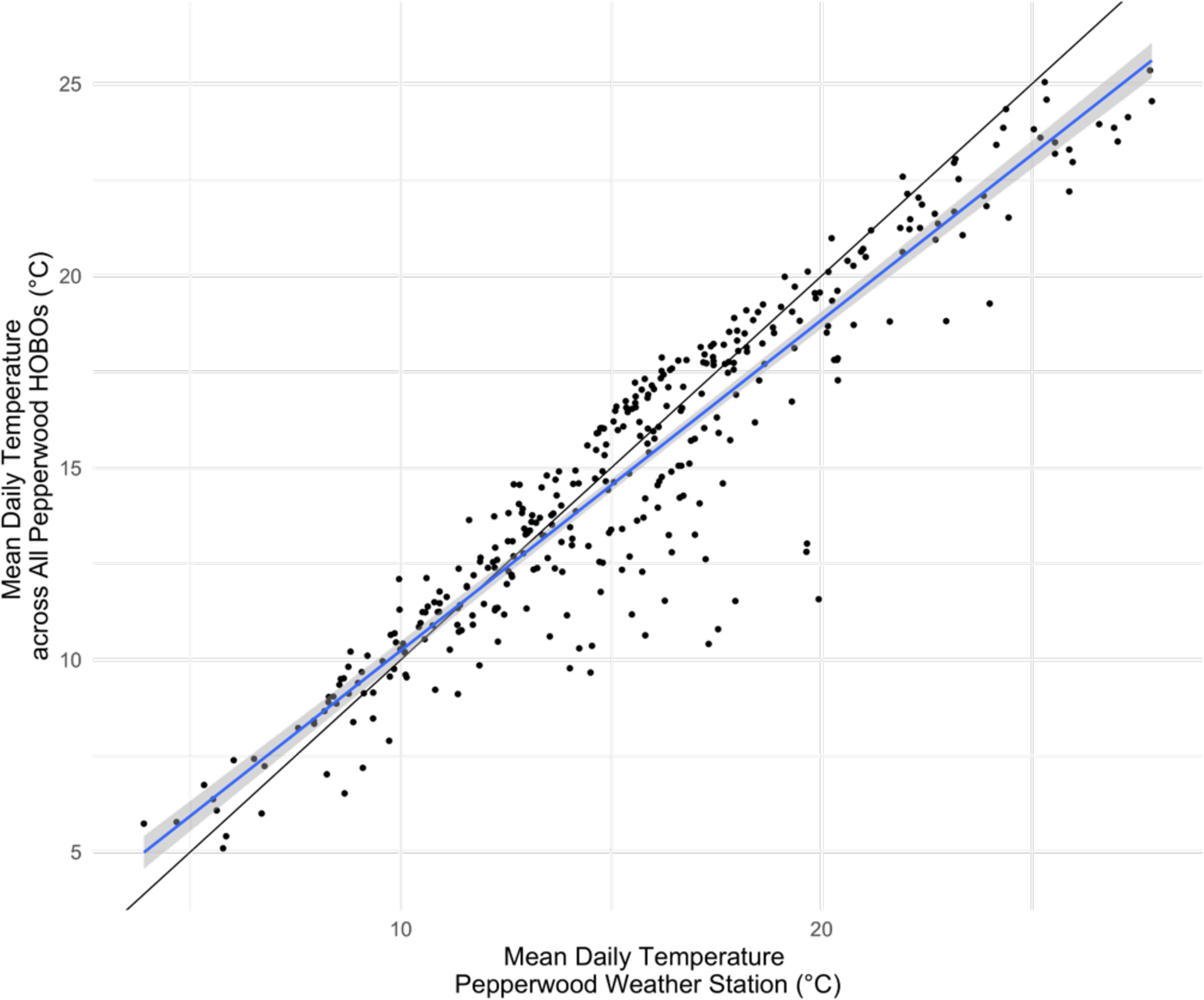

Supplement: S1 Appendix — Appendix A. Fig A.1. Fig A.1: Regression of climate variables against PC1 of physiographic space. Dominant variable explaining PC1 is DEM (elevation). Fig A.2. Fig A.2: Regression of climate variables against PC2 of physiographic space. Dominant variables explaining PC2 are canopy, PLP500 and northness. Fig A.3. Fig A.3: Custom radiation shield. Fig A.4. Fig A.4: Cold-air pooling evident on the northwest corner Pepperwood Preserve. (a) Mean minimum temperatures highlight inversion. (b) Season fluctuation (summer mean–winter mean) pronounced in the upper northwest corner of the Pepperwood Preserve, possibly part of larger cold-air pooling phenomena. Table. A.1. Table A.1 - RDA results showing climate space (only seasonal metrics) when constrained against physiographic space. Variables in bold are statistically significant (p < 0.05). Table. A.2. Table A.2 - Physiographic predictor variables and their effects. Appendix B. Fig B.1. Fig B.1: Mean temperature comparisons between HOBOs and an open site (Pepperwood Weather Station). Fig B.2. Fig B.2: Mean diurnal fluctuation comparisons between HOBOs and an open site (Pepperwood Weather Station) colored by elevation. Fig B.3. Fig B.3: Average annual temperature of each HOBO against the elevation (R2 = 0.23, p-value < 0.001). Fig B.4. Fig B.4: Pepperwood Preserve with 50 study sites numbered. (ZIP) [file pone.0300378.s001.zip › FigB1.tif]

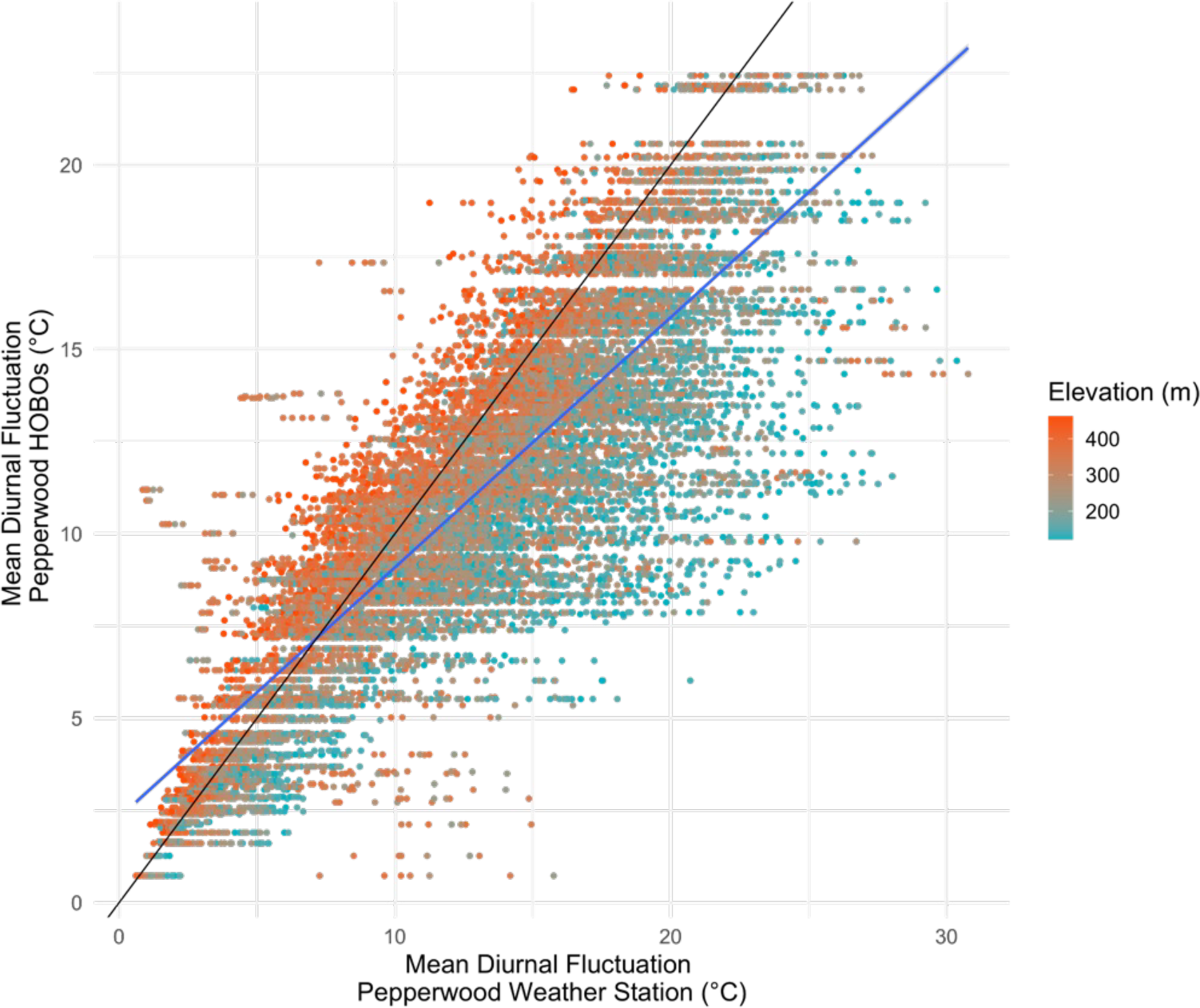

Supplement: S1 Appendix — Appendix A. Fig A.1. Fig A.1: Regression of climate variables against PC1 of physiographic space. Dominant variable explaining PC1 is DEM (elevation). Fig A.2. Fig A.2: Regression of climate variables against PC2 of physiographic space. Dominant variables explaining PC2 are canopy, PLP500 and northness. Fig A.3. Fig A.3: Custom radiation shield. Fig A.4. Fig A.4: Cold-air pooling evident on the northwest corner Pepperwood Preserve. (a) Mean minimum temperatures highlight inversion. (b) Season fluctuation (summer mean–winter mean) pronounced in the upper northwest corner of the Pepperwood Preserve, possibly part of larger cold-air pooling phenomena. Table. A.1. Table A.1 - RDA results showing climate space (only seasonal metrics) when constrained against physiographic space. Variables in bold are statistically significant (p < 0.05). Table. A.2. Table A.2 - Physiographic predictor variables and their effects. Appendix B. Fig B.1. Fig B.1: Mean temperature comparisons between HOBOs and an open site (Pepperwood Weather Station). Fig B.2. Fig B.2: Mean diurnal fluctuation comparisons between HOBOs and an open site (Pepperwood Weather Station) colored by elevation. Fig B.3. Fig B.3: Average annual temperature of each HOBO against the elevation (R2 = 0.23, p-value < 0.001). Fig B.4. Fig B.4: Pepperwood Preserve with 50 study sites numbered. (ZIP) [file pone.0300378.s001.zip › FigB2.tif]

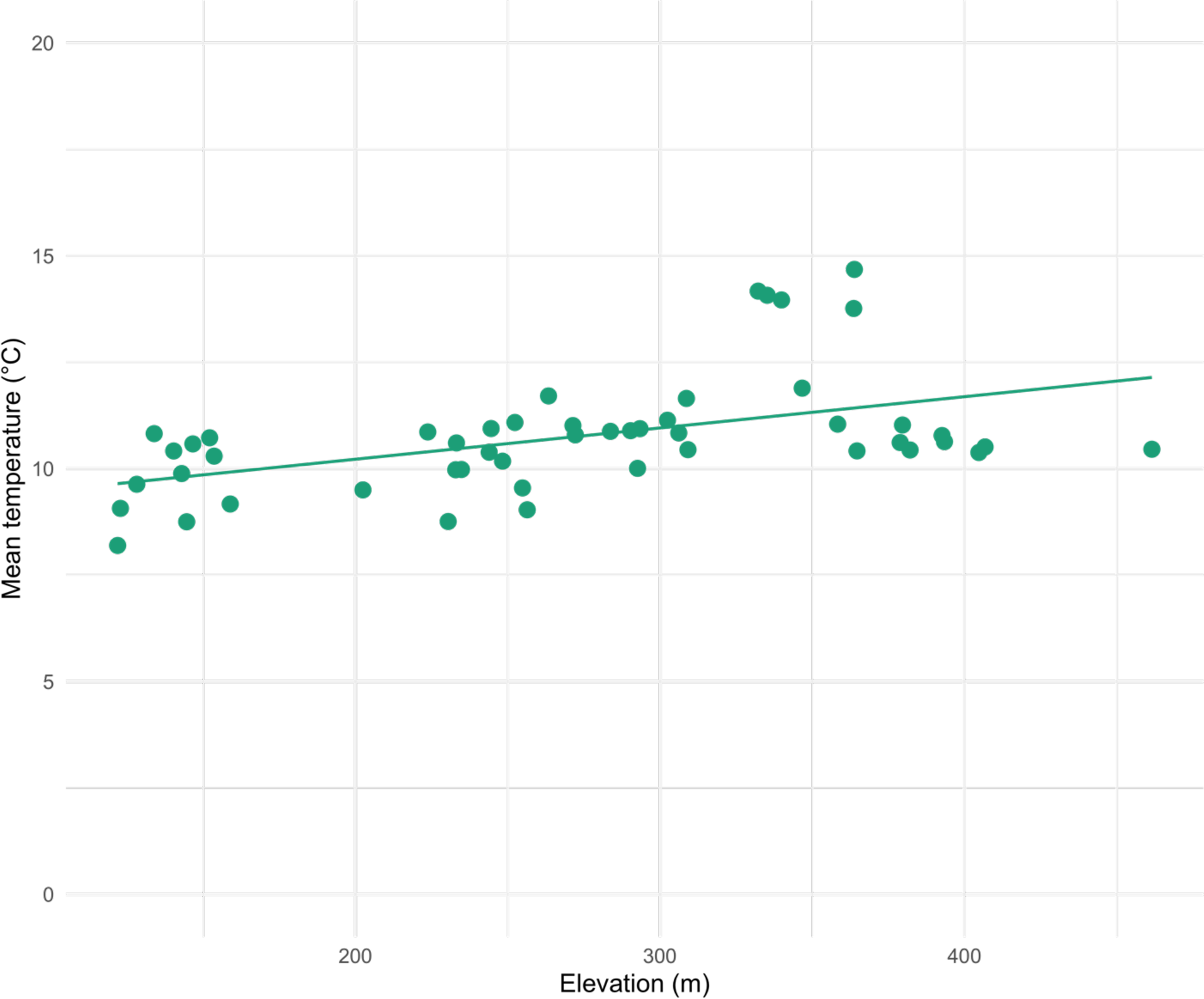

Supplement: S1 Appendix — Appendix A. Fig A.1. Fig A.1: Regression of climate variables against PC1 of physiographic space. Dominant variable explaining PC1 is DEM (elevation). Fig A.2. Fig A.2: Regression of climate variables against PC2 of physiographic space. Dominant variables explaining PC2 are canopy, PLP500 and northness. Fig A.3. Fig A.3: Custom radiation shield. Fig A.4. Fig A.4: Cold-air pooling evident on the northwest corner Pepperwood Preserve. (a) Mean minimum temperatures highlight inversion. (b) Season fluctuation (summer mean–winter mean) pronounced in the upper northwest corner of the Pepperwood Preserve, possibly part of larger cold-air pooling phenomena. Table. A.1. Table A.1 - RDA results showing climate space (only seasonal metrics) when constrained against physiographic space. Variables in bold are statistically significant (p < 0.05). Table. A.2. Table A.2 - Physiographic predictor variables and their effects. Appendix B. Fig B.1. Fig B.1: Mean temperature comparisons between HOBOs and an open site (Pepperwood Weather Station). Fig B.2. Fig B.2: Mean diurnal fluctuation comparisons between HOBOs and an open site (Pepperwood Weather Station) colored by elevation. Fig B.3. Fig B.3: Average annual temperature of each HOBO against the elevation (R2 = 0.23, p-value < 0.001). Fig B.4. Fig B.4: Pepperwood Preserve with 50 study sites numbered. (ZIP) [file pone.0300378.s001.zip › FigB3.tif]

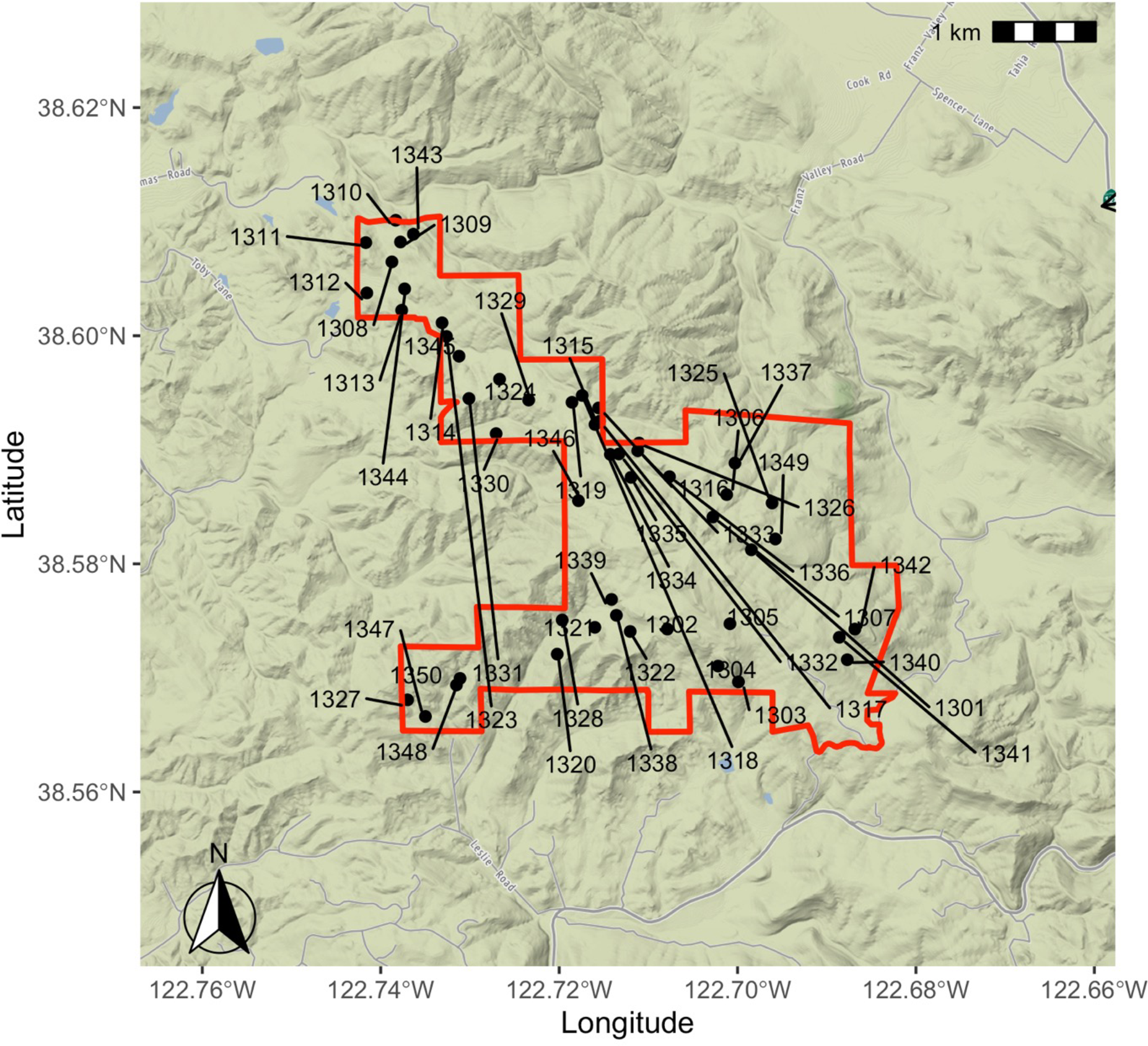

Supplement: S1 Appendix — Appendix A. Fig A.1. Fig A.1: Regression of climate variables against PC1 of physiographic space. Dominant variable explaining PC1 is DEM (elevation). Fig A.2. Fig A.2: Regression of climate variables against PC2 of physiographic space. Dominant variables explaining PC2 are canopy, PLP500 and northness. Fig A.3. Fig A.3: Custom radiation shield. Fig A.4. Fig A.4: Cold-air pooling evident on the northwest corner Pepperwood Preserve. (a) Mean minimum temperatures highlight inversion. (b) Season fluctuation (summer mean–winter mean) pronounced in the upper northwest corner of the Pepperwood Preserve, possibly part of larger cold-air pooling phenomena. Table. A.1. Table A.1 - RDA results showing climate space (only seasonal metrics) when constrained against physiographic space. Variables in bold are statistically significant (p < 0.05). Table. A.2. Table A.2 - Physiographic predictor variables and their effects. Appendix B. Fig B.1. Fig B.1: Mean temperature comparisons between HOBOs and an open site (Pepperwood Weather Station). Fig B.2. Fig B.2: Mean diurnal fluctuation comparisons between HOBOs and an open site (Pepperwood Weather Station) colored by elevation. Fig B.3. Fig B.3: Average annual temperature of each HOBO against the elevation (R2 = 0.23, p-value < 0.001). Fig B.4. Fig B.4: Pepperwood Preserve with 50 study sites numbered. (ZIP) [file pone.0300378.s001.zip › FigB4.tif]

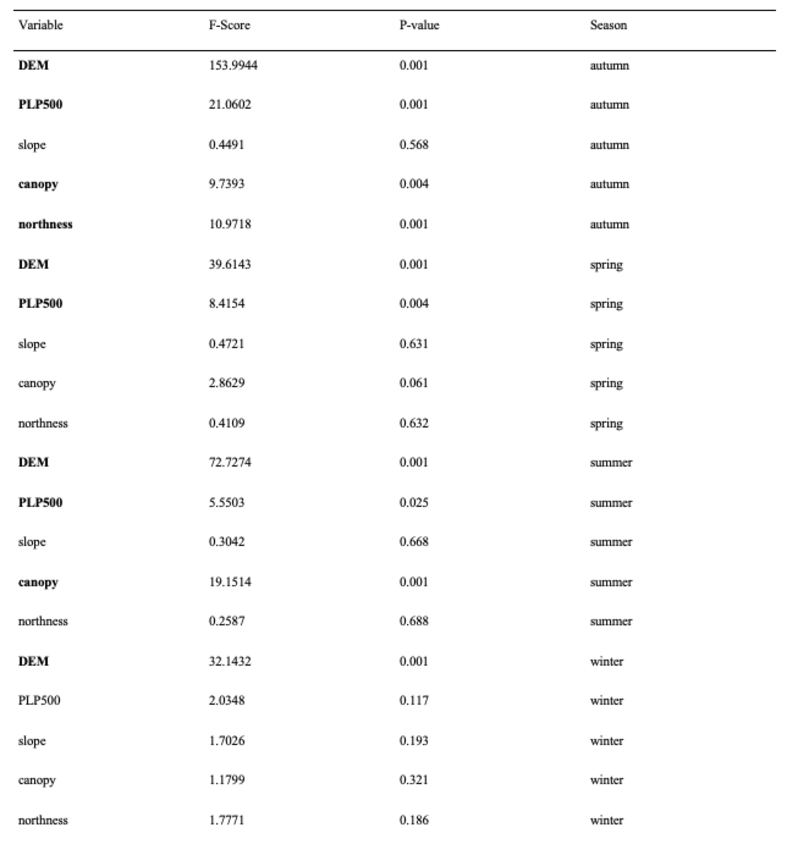

Supplement: S1 Appendix — Appendix A. Fig A.1. Fig A.1: Regression of climate variables against PC1 of physiographic space. Dominant variable explaining PC1 is DEM (elevation). Fig A.2. Fig A.2: Regression of climate variables against PC2 of physiographic space. Dominant variables explaining PC2 are canopy, PLP500 and northness. Fig A.3. Fig A.3: Custom radiation shield. Fig A.4. Fig A.4: Cold-air pooling evident on the northwest corner Pepperwood Preserve. (a) Mean minimum temperatures highlight inversion. (b) Season fluctuation (summer mean–winter mean) pronounced in the upper northwest corner of the Pepperwood Preserve, possibly part of larger cold-air pooling phenomena. Table. A.1. Table A.1 - RDA results showing climate space (only seasonal metrics) when constrained against physiographic space. Variables in bold are statistically significant (p < 0.05). Table. A.2. Table A.2 - Physiographic predictor variables and their effects. Appendix B. Fig B.1. Fig B.1: Mean temperature comparisons between HOBOs and an open site (Pepperwood Weather Station). Fig B.2. Fig B.2: Mean diurnal fluctuation comparisons between HOBOs and an open site (Pepperwood Weather Station) colored by elevation. Fig B.3. Fig B.3: Average annual temperature of each HOBO against the elevation (R2 = 0.23, p-value < 0.001). Fig B.4. Fig B.4: Pepperwood Preserve with 50 study sites numbered. (ZIP) [file pone.0300378.s001.zip › TableA1.tif]
