# Supplementary material for: Topography influences diurnal and seasonal microclimate fluctuations in hilly terrain environments of coastal California
Source: PLoS One. 2024 Mar 29;19(3):e0300378. doi: 10.1371/journal.pone.0300378 (PMC10980203; doi:10.1371/journal.pone.0300378)
Supplement: Attachment — Submitted filename: Review PLOS.pdf [file pone.0300378.s002.pdf]

Title: Topography influences diurnal and seasonal microclimate fluctuations in hilly terrain environments of coastal California

The article investigates the influence of topography and canopy cover on microclimate fluctuations in hilly terrain environments of coastal California using multiple linear regression and multivariate techniques. For the 50 sites representing the diverse topography and canopy cover, the role of 18 climate variables (related to temperature), 5 topographic and canopy related variables on the diurnal fluctuation and seasonal extreme temperature is studied. Though only a year of data is used to conclude the influence of the dominant variables on seasonal fluctuations, it is clearly written in the limitation of the article. Therefore, the paper is informative and contains findings that could warrant its publication.

I have some minor suggestions in the manuscript.

1. The recent literatures related to the work needs to be included in the introduction section.
2. Figure 2: The study site numbering must be shown in the Figure 2.
3. Table 1 and Table 2: the spacing is missing between the values in table. Ex: 11.56,19.75 (15.96) space missing
4. L188: Two sites (Site 1348 and 1350) were missing – Though the site numbering is mentioned its location is details are not shown anywhere.
5. Table 3 limit the significant digits in the partial coefficients value of different climatic variables to 2 or 3.
6. Redundancy analysis in short RDA is already mentioned in line no 204, no need to mention again in line no 292.
7. L296-298, "In autumn, topographic features (DEM, PLP500, northness) and canopy are found to be statistically significant (p-value < 0.005), but in spring, topographic features DEM and northness are significant (p-value < 0.005)". The sentence is not clear. Also, the significant variable in spring is DEM and PLP500 as per the Table A1 not the northness.
8. L321-326 , L328-331: The significance level (p-value < 0.001) is shown for every R^2 value. As all the values area significant at same significance level, it can be written only once at the end.
   Ex: Autumn (R^2 = 65%), Spring (R^2 = 28%), Summer (R^2 = 41%), and Winter (R^2 = 58) all significant at p-value < 0.001.
