# Supplementary material for: Topography influences diurnal and seasonal microclimate fluctuations in hilly terrain environments of coastal California
Source: PLoS One. 2024 Mar 29;19(3):e0300378. doi: 10.1371/journal.pone.0300378 (PMC10980203; doi:10.1371/journal.pone.0300378)
Supplement: Attachment — Submitted filename: PLOS One - Response Letter.pdf [file pone.0300378.s004.pdf]

Dear Editor,

It is our pleasure to submit the attached revision of "Topography influences diurnal and seasonal microclimate fluctuations in hilly terrain environments of coastal California" for publication in PLoS One. We were very pleased to receive the positive comments from three reviewers and welcomed the opportunity to provide additional information and clarify our presentation. We have now carefully addressed all the comments (see below).

Thank you for considering our re-submission and look forward to hearing the outcome. Our responses are in BLUE.

Reviewer #1: The article investigates the influence of topography and canopy cover on microclimate fluctuations in hilly terrain environments of coastal California using multiple linear regression and multivariate techniques. For the 50 sites representing the diverse topography and canopy cover, the role of 18 climate variables (related to temperature), 5 topographic and canopy related variables on the diurnal fluctuation and seasonal extreme temperature is studied. Though only a year of data is used to conclude the influence of the dominant variables on seasonal fluctuations, it is clearly written in the limitation of the article. Therefore, the paper is informative and contains findings that could warrant its publication.

Thank you for supporting our research, and we sincerely value your review.

I have some minor suggestions in the manuscript.

1. The recent literatures related to the work needs to be included in the introduction section.

We have revised the Introduction section now, please see following lines

*"..by e.g., weather station (Ashcroft et al. 2012, De Frenne et al. 2021). Subsequently, buffering capacity, in the context of our study, refers to the ability of a specific location to moderate or lessen temperature fluctuations (Frey et al. 2016, Gril et al. 2023). When a location has a high buffering capacity, it can effectively reduce the impact of temperature variations, providing more stable and moderated temperature conditions. Alternatively, a reduced buffering capacity implies that the location is less effective at mitigating temperature fluctuations, resulting in more pronounced and variable temperature changes (Braem et al. 2023)."*

*"We also revealed that canopy mediates the diurnal variation in different seasons (Gilbert et al. 2022)".*

*“Facilitated by unique micro-topographic characteristics (Dobrowski 2011, Patsiou et al. 2014), this can offer opportunities for species to persist in place, rather than shift in space, in response to climate change (Hampe and Petit 2005, Ashcroft et al. 2012, Lawler et al. 2015, Thurman et al. 2020)”*

*“...depressions and ridgetops, canopy cover, distance to coast, and proximity to the forest edge (Ewers and Banks-Leite 2013, Jucker et al. 2018, Lindenmayer et al. 2022)”*

*“...the resulting minimum and maximum extreme temperatures, are known to structure plant and animal distributions (Sexton et al. 2009, Lembrechts et al. 2019, Braem et al. 2023).”*

*“Buffering capacity, in the context of our study, refers to the ability of a specific location to moderate or lessen temperature fluctuations (Frey et al. 2016, Gril et al. 2023).”*

*“Alternatively, a reduced buffering capacity implies that the location is less effective at mitigating temperature fluctuations, resulting in more pronounced and variable temperature changes (Braem et al. 2023). “*

2. Figure 2: The study site numbering must be shown in the Figure 2.

Thank you for the suggestion, We have now included a new figure of the study site with numbers in Appendix (Figure B.4). We chose not to alter the original figure to avoid clutter, as including site numbers would have made the visualization complex.

3. Table 1 and Table 2: the spacing is missing between the values in table. Ex: 11.56,19.75 (15.96) space missing

Thank you for pointing this out, we have formatted the table values

4. L188: Two sites (Site 1348 and 1350) were missing – Though the site numbering is mentioned its location is details are not shown anywhere.

Please see the appendix figure B.4, it's on the lower left of the Pepperwood Preserve.

5. Table 3 limit the significant digits in the partial coefficients value of different climatic variables to 2 or 3.

Thank you for the suggestion, we have formatted the values to two significant digits.

6. Redundancy analysis in short RDA is already mentioned in line no 204, no need to mention again in line no 292.

Thank you for the suggestion, we have edited the line.

7. L296-298, “In autumn, topographic features (DEM, PLP500, northness) and canopy are found to be statistically significant (p-value < 0.005), but in spring, topographic features DEM and northness are significant (p-value < 0.005)”. The sentence is not clear. Also, the significant variable in spring is DEM and PLP500 as per the Table A1 not the northness.

We have rephrased it for clarity, thank you for pointing it out. Now the line reads ‘"*During autumn, statistical significance (p-value < 0.005) is observed for topographic features such as DEM, PLP500, and northness, as well as canopy. However, in spring, only topographic features DEM and northness demonstrate significance (p-value < 0.005).*"

The reviewer's observation is accurate. In our previous version, we inadvertently mentioned "northness" instead of "PLP500," alongside DEM, as the significant variable in spring. This error has been rectified in the revised version.

8. L321-326 , L328-331: The significance level (p-value < 0.001) is shown for every R2 value. As all the values area significant at same significance level, it can be written only once at the end.
Ex: Autumn (R2 = 65%), Spring (R2 = 28%), Summer (R2 = 41%), and Winter (R2 = 58) all significant at p-value < 0.001.

We agree with the reviewer, and have rephrased it, now, the lines read as follows - *Autumn ($R^2$ = 0.65), Spring ($R^2$ = 0.28), Summer ($R^2$ = 0.41), and Winter ($R^2$ = 0.58) all significant at p-value < 0.001;....Autumn ($R^2$ = 0.31), Spring ($R^2$ = 0.31), Summer ($R^2$ = 0.45) significant at p-value <0.001, and Winter ($R^2$ = 0.02), not significant at p-value = 0.31.*

Please also note that we have amended the $R^2$ as a proportion instead of %.

Reviewer #2: Based on the annual understory temperature changes of 50 surveyed locations, this study investigated the effects of terrain and tree cover on near surface temperature changes. The research results may help to improve our ecological understanding of fine-grained seasonal climate change in coastal environments. However, there are some concerns that the authors should address before it can be considered for publication.

Thank you for providing insightful comments to our manuscript, we have addressed the raised concerns and hope that the revisions meet your satisfaction.

1. In the last paragraph of the introduction, I suggest the authors further highlight the significance of this study.

Thank you for the suggestion, we now state the significance of our study as *“Knowing how species respond to changes in diurnal temperature fluctuations is crucial for understanding their adaptation to climate (Davis et al. 2005).”*

2. In the data, the authors should add more information about data, such as DEM data availability and access.

Thanks for the suggestion, details about data can be found in these line *Spatial analysis using a GIS (Popescu and Gibbs 2010) was performed on a 10 m digital elevation model (DEM) of Pepperwood Preserve (Oldfather et al. 2016) to derive topographic variables (for details on topographic variables used in this study see Oldfather et al. 2016) (Table 2 and Table A.2).*

3. I suggest the authors add more descriptions of the climate in the study area, such as multi-year average annual temperature.

Thank you for the suggestion, we have added comparison of study site averages with the regional, please see the following *The long-term average temperature at the study site was found to be 15.37 °C, with a standard deviation of 0.53 °C. In contrast, the regional temperature is found to be 14.49 °C with a standard deviation of 0.55 °C. These values signify the variability in temperature over the past 40 years.*

4. Lines 188-190, the authors mentioned that meteorological stations 1348 and 1350 have missing values. How did the author handle the missing values?

We utilized the available data for those two sites. We suspect that the reviewer may be interested in whether the outcome differed depending on whether the missing data was interpolated or if partial data was used for both the sites. We conducted the analysis by retaining the incomplete data for the two sites and also by completely removing it. In both cases, the

results remained unaffected. We clarified it by adding explicitly the following *"Two sites (Site 1348 and 1350) were missing climate data for roughly six months , but were still included in all the analyses for completeness, and removing or including them did not alter the outcome."*.

5. Lines 213-216, the authors analyzed the average temperature of 55 stations in the study area, but this cannot reflect the impact of altitude on temperature. I suggest the authors partition the site based on altitude and compare the temperatures of different altitude sites.

We acknowledge the potential influence of altitude on temperature, but our limited sample precludes a partitioning of the data. However, we now report the relationship between average temperature and altitude in the manuscript. Please see the following text *"Across the landscape, altitude was positively correlated with average temperature (Fig. B.3; $R^2 = 0.23$, $p < 0.001$)."* and new appendix figure (Fig B.3).

6. More mechanistic explanations should be added to further explain the relationship between altitude and temperature changes.

Thank you for the suggestion, we have added several mechanistic explanations that cover orographic effects, solar radiation and localized climate variations. Please see following lines

*Generally, the relationship between temperature and elevation (or altitude) is explained by the adiabatic lapse rate of 6-8°C decrease per 1000 meters increase in elevation. However, ridgetops and valley bottoms can influence temperatures via orographic effects, causing adiabatic cooling on windward slopes and potential warming on leeward sides.*

*During the winter months, when the sun's angle is lower, there is reduced direct sunlight compared to the higher sun angles experienced in summer. This difference in sunlight exposure is likely a key factor explaining the canopy effects observed exclusively during the summer season. These effects are pronounced due to variations in canopy cover, with summer showing a greater seasonality because of the trees providing more effective shading during this period (Geiger and Bouyoucos 1951, Estevo et al. 2022).*

7. A paragraph of limitation discussion should be added to clarify the limitation or uncertainty of data and methods in this current study. For example, uneven distribution of meteorological stations (Shen et al., 2014, 2018; Wang et al., 2016) may affect the research results.

Thank you for the suggestion, we have added thee following to highlight additional limitations in our study, *"Another point of caution relates to the potential uncertainties in the collected data due to the stratification of temperature loggers as the readings may be influenced by environment around the loggers, such as shading from canopy or being in open (Shen et al. 2014, 2018, Wang et al. 2016). Additionally, the use of radiation shields can introduce bias in*

*temperature readings, emphasizing the importance of being mindful of the type of radiation shielding employed (Maclean et al. 2021).”*

References:

Spatiotemporal change of diurnal temperature range and its relationship with sunshine duration and precipitation in China. Journal of Geophysical Research: Atmospheres, 2014, 119: 13163-13179.

Weak cooling of cold extremes versus continued warming of hot extremes in China during the recent global surface warming hiatus. Journal of Geophysical Research: Atmospheres, 2018, 123: 4073-4087.

Wang W, Lu H, Yang D, et al. Modelling hydrologic processes in the Mekong River Basin using a distributed model driven by satellite precipitation and rain gauge observations. PloS one, 2016, 11(3): e0152229.

Reviewer #3
Line - 113
I am having difficulties following you argumentation here. If you define buffering "as reduced temperature fluctuations relative to free atmospheric conditions" in line 72/73 and state at the beginning of this sentence that valley bottoms will show greater temperature fluctuations - where does the reduced buffering capacity come from? Please clarify!

We have provided clarifications now, please see lines

*“Buffering capacity, in the context of our study, refers to the ability of a specific location to moderate or lessen temperature fluctuations (Frey et al. 2016, Gril et al. 2023). When a location has a high buffering capacity, it can effectively reduce the magnitude of temperature variations, providing more stable and moderated temperature conditions. Alternatively, a reduced buffering capacity implies that the location is less effective at mitigating temperature fluctuations, resulting in more pronounced and variable temperature changes (Braem et al. 2023). “*

Figure 1
I'd suggest to add an arrow head to the temperature axis to illustrate that this is a continuous variable (different from your x-axis)

make sure to explain the X (seasonal mean, I guess?)

Using simply the term elevation here might be too simplistic - it's rather the relative elevation of the site compared to its surroundings than the absolute elevation - it gets clearer as you name rides and valleys, but I would suggest to be more precise here. Would "topographically exposed" versus "topographically sheltered" work? - I know, not as catchy as elevation, but more correct in terms of content ...

Thank you for the suggestions, we have modified Figure 1. The continuous annotation for Temperature was very crucial, and we have given the context to elevation - incorporated ‘Topographically Sheltered’ and ‘Topographically Exposed’ into the figure.

Line 148
regional what - seems like a word is missing here

Thanks for pointing out the missing word, we have corrected it now, it reads “*To evaluate coupling and buffering of a site with regional climate (in this case. we used a weather station)..*”

Line 158
please cite (Use of GIS) and ‘10 m digital elevation model (DEM)’ please provide the source

We have cited the use of GIS as " …*GIS (Popescu and Gibbs 2010)*" and cited the source of the DEM "..*(Oldfather et al. 2016)*"

Line 164
Could you provide any reasoning why exactly 100 and 500 meters?

The 100-meter radius is likely to capture fine-scale variations, whereas the 500-meter radius is better suited for delineating larger terrain characteristics. We added the following to provide clarity *"100 m and 500 m radii were employed to capture fine-scale variations and delineate larger terrain features. "*

Line 168
instead of the TPI variables, which were not used at all? What were the criteria to go for PLP500 instead of PLP100?

As PLP was strongly correlated with TPI, we used PLP. In a prior study (Oldfather et al. 2016*)* that used some of these variables for evaluating relationship  between topographic complexity and species distribution found that the metrics calculated with radius of 500 m were similar to 100 m. Hence, we used 500 m only. We have addressed this in the following lines *"PLP and TPI measures at each scale were strongly correlated (pairwise $r^2$ ranged from .84 to .94), hence PLP500 was selected for use in this study. Furthermore, the metrics calculated using 500 m were similar to 100 m, so only PLP500 was used in all the analyses (Oldfather et al. 2016)."*
Line 169
please cite appropriately - both the package and R!

We have provided proper citations now.

Line 171-172
How does this fit with your statement in line 167/168? OK, reading it over again, it seems like you have used PLP500, but none of the two TPI variables? This could be made a touch clearer.

We have now clarified the statement,  please see the lines *"PLP and TPI measures at each scale were strongly correlated (pairwise $r^2$ ranged from .84 to .94), hence PLP500 was selected for use in this study. Furthermore, the metrics calculated using 500 m were similar to 100 m, so only PLP500 was used in all the analyses (Oldfather et al. 2016)."*

Line 176-177
Be aware that such an approach is likely to give misleading results: If you have a total cover of let's say 80%, possibly composed of 60% deciduous and 40% evergreen, you would get 20% instead of 40% with your calculation! Please clarify!

Apologies for the confusion. Our approach was to subtract the fraction of deciduous cover from the total canopy cover, hence, we have rephrased the main text to state: "*Canopy cover for winter was modified by subtracting the deciduous fraction from the total canopy cover.*"

Line 191
apart from the already mentioned raster package, please make sure to cite all additional packages you have used (like probably vegan?)

Thank you for the suggestion, we have now included the major pancakes that were used. We java now included the additional packages, and statement now reads "All analyses were performed using R software (R Core Team 2021) using several R packages, including '*tidyverse'*, '*raster*' and '*vegan*'"

Line 260-261
What do you mean by this sentence? Reads like you are using different scales along the axes, are those distinguished within the diagrams?

Apologies for the confusing statement in the caption. We have revised the caption as follows" *"Blue dots on the panels represent sites, and blue arrows represent variables, with their length indicating their contribution to the principal component."*

Line 273
it is likely a matter of taste, but expressing R2 as a percentage is not too common

We have now revised the entire manuscript to use proportion instead of percentage.

Figure 5
I'd suggest to order the legend entries the other way around, similar to the coloring of the diagram

Thank you for the suggestion. We have now revised the plot, aligning the order of legend entries with the color ramp.

Line 311
Reading "slope" in the context of lower elevation sites and higher elevation sites, one ultimately thinks about topographic slope, not the slope of the linear fit shown in Fig. 6!

Thank you for pointing out the possible confusion, we have revised the sentence now to say '*especially in winter as evidenced by the positive association of minimum temperatures with elevation*.'

Line 321
instead of the R2 value, I'd suggest to provide the slope together with the p-value (using the asterisk-notation throughout the text, rather than writing always p<0.001)

We have fixed the notations to be a correlation coefficient as proportion.

Line 323
Please check! Based on the figure and the provided statistics, I would say there's a clear trend in summer max with elevation! Do you mean winter?

The reviewer is correct, we wanted to say that in contrast to winter, we have amended the text to say *"Summer maximum temperatures are found to be declining with elevation, but are less pronounced in winter."*

Line 324
please check the wording!

Apologies for the confusing wording. The revised text now states: "*..maximum temperatures association with elevation in autumn are found to be less pronounced compared to spring*."

Fig 7A
When talking about temperature variation, I would go for Kelvin (K) as unit rather than degrees Celsius.

We appreciate reviewers suggestion, as in either case the values would not be negative and would start from absolute zero (implying no fluctuation where min and max temperature are the same for a day), we feel Celsius would be a better choice. Additionally, we have used Celsius in all our analysis, it would be easier to communicate with the reader.

Fig 7B
I may have missed it - what do you mean by mean seasonal variation here if it is not differentiated into seasons?

We apologize for the confusion, it is the difference between summer and winter mean. We have added a clarification now' *"...lower seasonal variation (difference between mean summer and winter fluctuation)."*

Line 360-362
Do they really do? If so, you could actually stop writing here ;)
Isn't it more like you state in your conclusion: "we confirm the role of elevation and suggest that interactions with finer physiographic features would be key"?

We agree with the reviewer that we may have oversimplified our main takeaway. Indeed, temperature variations are dependent on physiographic features. We have now provided clarification by stating: *"Given that our results align with general understanding of elevation as the key factor in temperature, its interaction with more detailed physiographic features influence temperature variation (Dobrowski 2011, Marsh et al. 2022). This supports the validity of*

*downscaling approaches that use coarse-grain climate grids to produce fine-grained products using only the elevation (Ashcroft 2010, Davy and Kusch 2021)".*

Line 371-372
please check the content/structure/wording - it looks like exposed sites are more coupled to free-air conditions (Fig. B.2) than sheltered sites?

Thank you for pointing out the wording error, we have now edited it as follows *"Furthermore, in our study site, we find that the more exposed a site is, like the ridgetops, the fluctuations are more coupled to free-air temperature than the valley bottom sites that tend to be lower."*

**References**

Marsh, C., D. Krofcheck, and M. D. Hurteau. 2022. Identifying microclimate tree seedling refugia in post-wildfire landscapes. Agricultural and Forest Meteorology 313:108741.

Oldfather, M. F., M. N. Britton, P. D. Papper, M. J. Koontz, M. M. Halbur, C. Dodge, A. L. Flint, L. E. Flint, and D. D. Ackerly. 2016. Effects of topoclimatic complexity on the composition of woody plant communities. AoB Plants 8:plw049.
